# Coherence loss of partially mode-locked fibre laser

**DOI:** 10.1038/srep24995

**Published:** 2016-04-29

**Authors:** Lei Gao, Tao Zhu, Stefan Wabnitz, Min Liu, Wei Huang

**Affiliations:** 1 Key Laboratory of Optoelectronic Technology & Systems (Ministry of Education), Chongqing University, Chongqing 400044, China; 2 Dipartimento di Ingegneria dell’Informazione, Università degli Studi di Brescia and INO-CNR, via Branze 38, 25123 Brescia, Italy

## Abstract

Stochastically driven nonlinear processes limit the number of amplified modes in a natural system due to competitive mode interaction, which is accompanied by loss of coherence when increasing the complexity of the system. Specifically, we find that modulation instability, which exhibits great fluctuations when it spontaneously grows from noise in conservative systems, may possess a high degree of coherence in dissipative laser system with gain. Nonlinear mode interactions can be competitive or cooperative: adjusting the intracavity polarization state controls the process of loss of coherence. Single-shot spectra reveal that, first, the fibre laser redistributes its energy from the center wavelength mode into sidebands through parametric instabilities. Subsequently, longitudinal modes are populated via cascaded four-wave-mixing. Parametric frequency conversion populates longitudinal modes with a random distribution of position, intensity and polarization, resulting in partially (rather than highly) coherent pulses. These dynamics unveil a new route towards complex pattern formation in nonlinear laser systems, and they may be also beneficial for the understanding of supercontinuum, Kerr-combs phenomena, and optical rogue waves.

A transition from highly coherent to weakly coherent states is common in the nonlinear systems observed in different physical settings, such as optics^1^,^2^, Bose-Einstein condensation^3^, semiconductors^4^, free-electron lasers^5^, and fluids^6^. For specific conservative and dissipative systems, stable coherent solutions with self-sustaining properties can be observed. For example, solitons are formed owing to a balance between nonlinearity and diffraction/dispersion in Hamiltonian systems; dissipative solitons occur under the additional balance of loss and gain^7^,^8^,^9^. Soliton systems possess a high degree of coherence, owing to well-determined phases among their different longitudinal (or frequency) modes. However, spontaneous or noise driven processes are accompanied by an energy redistribution into additional modes, leading to a loss of coherence, that may eventually disrupt coherent soliton-like solutions. For example, consider modulation instability (MI), a process that exponentially amplifies noise upon propagation under the combined action of nonlinearity and dispersion/diffraction^10^,^11^,^12^,^13^. Owing to their stochastic origin, competitive mode interactions enhance fluctuations in single-shot spectra, until complete incoherence is found in both Stokes and anti-Stokes regions^10^.

In dissipative systems such as lasers, pattern formation may be initiated by the coherent excitation of MIs. When multi-wave mixing cascades among the MI gain bands, a pulsed laser output with partial coherence is observed. The output from such a partially mode-locked laser (PML) is sometimes referred to as noise-like pulses, since laser pulses *seem* to originate from the amplification of noise: bunches of pulses with irregular varying time duration and intensity are observed^14^,^15^,^16^,^17^,^18^,^19^. The autocorrelation trace of a PML contains a coherence peak of femtosecond duration, sitting on a broad pedestal with a duration ranging from several to hundreds of picoseconds. Moreover, the optical bandwidth of a PML is often comparable to or even larger than the gain bandwidth of the active medium^15^. As it represents a non-stationary state in a complex dissipative nonlinear system, understanding the underlying physics of PML is scientifically important but technically challenging. Although several experiments have investigated different properties (e.g., stochasticity, optical rogue waves, turbulence, and periodicity) of PMLs, their underlying physics still remains largely controversial^10^,^16^,^17^,^18^,^19^. The main reason for that is the presence of several competing nonlinear processes, which make it extremely difficult to observe the dynamics of PML formation. Besides, detecting optical spectra at MHz frequencies is technically challenging, as it requires the application of dispersive Fourier transformation (DFT), a technique which enables transient spectrum detection^10^. Different interpretations of PMLs have been proposed, for example: cavity nonlinear transmission and walk-off between two polarizations^14^, the combined effect of soliton collapse and positive cavity feedback^20^, and the oversaturation of the intracavity saturable absorber (SA)^21^. In this work we experimentally prove that PML action may originate from vector parametric frequency conversion, and that the mechanism of coherence loss is can be controlled by intracavity polarization rotation.

The mechanism of coherence loss in a PML is similar to the generation of chaotic Kerr combs based on parametric frequency conversion within high quality factor (Q) microresonators. In that context, both highly and partially coherent pulses can be produced, based on cascaded four-wave-mixing (FWM) among parametric gain lobes^22^. An universal route was proposed for the loss of coherence in microresonator frequency combs, based on the competition between primary and secondary combs^23^. The main principle of cavity parametric frequency conversion is schematically shown in Fig. 1(a). Primarily, energy transfers from a continuous wave (CW) central mode into parametric or MI gain sidebands: the corresponding longitudinal modes are thus populated. MI is a degenerate FWM (DFWM) process, where two pump photons at frequency of ω_0_ are annihilated, and two new photons with Stokes or anti-Stokes frequencies (ω_mS_ and ω_mAS_) are simultaneously generated. Both energy conservation and phase-matching conditions are satisfied. In microresonators, the frequency spacing (say, Δ) between the center mode and the primary sidebands is much larger than the spacing between longitudinal modes (or cavity free-spectral range, say, δ). Next, cascaded FWM may occur between the pump and the different MI gain lobes. When the pump power is high enough, newly generated frequencies merge into a gap-free or continuum comb.

In the time domain, the coherence of the generated pulses depends on both the coherence of parametric gain lobes and the phase-matching condition of cascaded FWM. For microresonator Kerr-combs, adjacent longitudinal modes populated in the MI gain lobes are highly coherent. The coherence of the formed pulses is mainly determined by the appearance of a sequence of sub-combs. Whenever Δ is not an integer multiple of δ, sub-combs with different offset frequencies are generated, resulting in poorly coherent pulses^23^. Highly coherent pulses may be generated within a laser cavity incorporating a high finesse Fabry-Pérot interferometer or a high Q microresonator^22^,^24^,^25^. In a polarization-maintaining fibre laser cavity, scalar cascade FWM occurs. However, both scalar and vector FWM may be supported in non-polarization-maintaining cavity (such as our fibre ring laser), so that both PML and highly coherent pulses may be generated, depending on the orientation of intracavity polarization controllers (PCs). In general, vector FWM processes occur whenever the center mode has an elliptical polarization state. As a matter of fact, two vector FWM processes with cross-coupling between two polarization modes may occur, namely, polarization MI (PMI)-like and cross-phase MI (XPMI)-like, as depicted in the insets of Fig. 1(a) 26. These processes coexist with scalar FWM and compete with each other, until the winner dominates the whole parametric process. When compared with the scalar case, the phase-matching conditions of cascaded PMI-like or XPMI-like FWM are even more complex, and in most cases, partially coherent pulses rather than high coherent pulses are generated due to the loosely fixed phase relation between the cavity modes.

In optical fibres with a self-focusing Kerr coefficient, anomalous dispersion is required for the nonlinear phase-matching condition of scalar MI. However, the presence of periodically varying dispersion and loss/gain in a fibre ring resonator can lift this restriction, and a specific kind of MI, parametric instability (PI), appears^27^,^28^,^29^. In this case, the condition for quasi-phase-matching (QPM) of the nonlinear FWM process can be expressed as 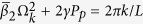, where, 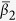 is the average dispersion, Ω_*k*_ is the pulsation detuning, *γ* is the nonlinear coefficient, *P*_*p*_ is the peak power in the quasi-CW mode, *L* is the cavity length or dispersion period, and *k* is the sideband order. Parametric frequency conversion occurs from the central mode into distant cavity modes such that the power-dependent QPM condition is satisfied. Figure 1(b) illustrates the numerically computed (see Methods section) PI gain within a range of peak powers, with parameters used in this experiment. We find two distinct spectral peaks, which agree quite well with the experimental results.

In this article, we experimentally demonstrate the formation process of PML based on PI and subsequent cascaded vector FWM among the PI gain lobes under cavity detuning control. The detuning can be freely changed by adjusting the state of polarization (SOP) of light passing through the SA, which exhibits a significant nonlinear phase response in our experiment. As we shall see, the dispersion-variation-induced PI that arises in the dissipative laser system exhibits little fluctuations, and quasi-periodic cooperative and competitive cascade FWM-activated mode interactions are obtained. We find that the polarization of each filtered wavelength of PML tends to dithering/vibrating, which indicates that the well-defined SOP, which is typical of dissipative solitons, is also broken in PML.

The fibre cavity is schematically shown in Fig. 2(a). The SA is fabricated by filling reduced graphene oxide (rGO) flakes into cladding holes of a photonic crystal fibre (PCF): more details are given in Supplementary 1. Since only a very small percentage (10^−7^) of light passing through the PCF will interact with rGO, the thermal damage threshold can be increased substantially, a situation which is inaccessible for conventional ferule methods^30^,^31^. Most importantly, the small nonlinear phase shift accumulated in each round trip makes it possible to observe the formation process of PML. The nonlinear transmission of the SA shown in Fig. 2(b) indicates a modulation depth of ~24%. The dispersion of 1 m EDF, 19.5 m DCF, and 14.5 m SMF are 15.7, −38, and 18 ps/nm/km, respectively, so that the net normal dispersion of the cavity is 0.737 ps^2^. Because of the relatively large polarization-dependent loss of graphene, biasing the intracavity PC varies the nonlinear phase response of our SA, as discussed in Supplementary 1. The nonlinear response of the SA and the intracavity dispersion variation are crucial in generating PI, as discussed in Supplementary 2.

Setting the power level of two pump lasers at 400 mW, and by carefully rotating the PC, we observed a stable PML regime with easiness. The typical oscilloscope trace contains no significant intensity fluctuations. The radio frequency spectrum exhibits a contrast ratio of 90 dB at the fundamental frequency of 5.82 MHz, suggesting that PML has very good stability. The autocorrelation trace in Fig. 2(c) exhibits a pedestal with a full width at half maximum (FWHM) of 30.5 ps, and the coherent peak is about 416 fs. The FWHM of the optical spectrum detected by a conventional OSA in Fig. 2(d) is 16 nm, and a good agreement is found between the 100 single-shot spectra detected by DFT and the time-averaged optical spectrum. The 6 consecutive single-shot spectra in Fig. 2(e) indicate the presence of large fluctuations, which are invisible in the averaged optical spectrum. The details of our measurements are described in the Methods section.

PML is obtained via rotating the PC, which permits to detune the nonlinear phase, and also to adjust the phase-matching condition of cascaded FWM. Figure 3 illustrates our results for different PC states, and with the fixed pump power of 800 mW. The averaged optical spectra in Fig. 3(a) suggest that at first the CW laser is destabilized by PI. Next, additional longitudinal modes are activated through cascaded FWM among different PI gain lobes, finally leading to PML by further optimizing the intracavity polarization evolution. The center mode and the four new sideband mode wavelengths, namely, λ_2AS_, λ_1AS_, λ_1S_, λ_2AS_, all satisfy the energy conservation of FWM. The PI sideband spacing is 0.438 THz, which is in relatively good agreement with the calculated value of Fig. 1(b) for the estimated intracavity peak power level of ~5W of the central mode quasi-CW pulses.

The corresponding single-shot spectra contain more information about the PML evolution process. Primarily, a stable and highly coherent pulse train is generated for PC state 1: a much broader optical spectrum is formed in the single-shot spectrum, and the corresponding highly coherent temporal outputs in Fig. 3(f) are generated. In this case, DFT automatically filters the giant CW mode component that does not participate to the pulse formation process. Considering that the resolution of the DFT is 0.2 nm (0.05 THz), the PI gain lobes contained in the single-shot spectrum are reliable. We notice that those parametric gain lobes come entirely from the CW state. A further rotation of the PC (from PC state 1 to 3) primarily leads to secondary PI gain lobes (Fig. 3(g–i)). As we can see, those consecutive single-shot spectra are rather uniform. During this process, energy is transferred from the center mode into the parametric gain lobes at distant wavelengths, and a giant envelope is gradually imposed on the pulse train (Fig. 3(f)). Continuously biasing the PC (from PC state 4 to 6) leads to supporting additional longitudinal modes, which eventually deplete the giant CW mode. The single-shot spectra appear as localized structures in two dimensions, as shown in Fig. 3(j–k). The spliced spectra in Fig. 3(e) for different round trips show that the well-defined structure in the averaged optical spectrum disappears in the single-shot spectrum. Finally, when the energy of the CW component is completely transferred into sidebands, the Q-switched-like envelope gradually diminishes, as more longitudinal modes are activated. Here, complete randomization is shown in its single-shot spectra, and a stable PML is formed.

Next we investigated the coherence of the PI gain lobes based on the statistical analysis of spectral correlations as described in ref. 10. The background-free correlations of the first Stokes region (0.9 THz) for different PC states are shown in Fig. 4. As can be seen, our results exhibit a great difference with respect to the case of MI in a conservative system^10^. Quasi-periodic fluctuations of the autocorrelation centered at zero indicate cooperative (positive value) and competitive (negative value) interactions of the individual cavity modes. Most importantly, both the positive center peak with zero frequency shift and the deep negative valley diminish gradually with polarization detuning. The same properties are observed in other Stokes and anti-Stokes regions. Yet, this correlation disappears when considering the whole spectrum (more details are given in Supplementary 3). When PML is formed, the autocorrelation trace of the first Stokes region finally evolves into to a flat zero-background, indicating that each mode is only correlated with itself. Such loss of correlation provides the representation of the loss of coherence.

Tuning of the pump strength may also form PML. Keeping the PC in state 7, we recorded the averaged optical spectra, temporal trace, and single-shot spectra for various levels of pump power. Figure 5(a) reveals that more longitudinal modes are activated when the pump strength grows larger: correspondingly, as shown in Fig. 5(b), irregular pulses with high intensities are formed. We notice that the temporal evolutions in Fig. 5 are highly different from what is observed when the PC detuning is changed. The temporal symmetry of leading and trailing pulse edges, which is apparent in Fig. 3(f), is absent when the pump power is varied. In this case the pulse intensity has a highly asymmetric temporal profile: it increases gently with an exponential trend in the leading pulse tail, while it decreases abruptly in the trailing edge.

The observed temporal asymmetry grows larger for stronger pumping levels. For low pump strengths, the pulse packets are localized between zero intensity regions (i.e., they are located in correspondence with the giant quasi-CW pulse). On the other hand, more packets appear when the pump power is high enough. For example, whenever the pump power is set at 750 mW, pulse packets merge with each other. Even higher pump powers flatten the pulse energy in each round-trip by coupling among different frequencies. Those properties clearly illustrate the onset of intracavity parametric frequency conversion, a nonlinear process that exhibits optical bistability when the phase matching is optimized. In fact, as shown by Fig. 5(c), phase-locking of longitudinal modes leads to a hysteresis loop for the average output power as the pump power is varied in opposite directions. The periodicity of the pulse packets leads to the appearance of discrete frequency components in the average optical spectra of Fig. 5(a), due to the limited detection time of our OSA.

Although the average optical spectra in Fig. 5(a) show that the giant quasi-CW mode remains present until the pump power is larger than 650 mW, the corresponding single-shot spectra reveal that the CW mode is depleted when the pump power is relatively small.

Figure 6(a) illustrates single-shot spectra for 100 mW pump power: as can be seen, quasi-CW and narrowband spectra are generated over the first few round trips, until new sidebands appear on both the Stokes and the anti-Stokes side after further laser circulations. The initial spectral broadening around the initial CW mode originates from self-phase modulation (SPM): here the spectrum remains relatively smooth until the generation of sidebands occurs. For round trip numbers larger than 40, the depletion of the quasi-CW region is clearly observed, in correspondence with the generation of the distant sideband frequencies. Here, only 1^st^ order Stokes and anti-Stokes sidebands are generated, and the depletion of the central quasi-CW pump mode region makes it hard to generate the 2^nd^ order Stokes and anti-Stokes sidebands. Pump mode depletion deteriorates the QPM phase matching condition of PI, and leads to an abrupt switching to CW lasing. This conclusion is not shown in the averaged optical spectra, yet it is in accordance with the simulation in Fig. 1(b), where only 1^st^ order sidebands are formed when the peak power is relative low.

The second characteristic is that, for relatively large pump powers, the CW mode quickly transfers its energy to high-order sidebands. This process is accompanied by a sudden evolution from a narrow quasi-CW spectrum into a set of broad but randomly distributed comb lines, as shown in Fig. 6(b). The multi-wave mixing process among the sidebands and central pumping region produces a randomized spectrum at each round trip. As the number of round trips necessary for accumulating a significant nonlinear phase shift decreases with increasing pump strength, the larger the pump power, the quicker the generation of pulse clusters. When the pump power is large enough, a broad comb with randomly spaced lines can be generated even within a single round trip.

In a PML based on the parametric frequency conversion process, different sets of longitudinal modes correspond to different portions of the pulse packets. To show this, we filtered out the PML output spectrum by a wavelength tunable filter with a bandwidth of 1 nm, and Fig. 7(a) depicts the filtered spectra. The corresponding temporal signals are shown in Fig. 7(b). It is clear that the center wavelength region (3), where SPM is the dominant effect, is responsible for the generation of the dense pulse train. For wavelengths far away from the center, the number of pulses in each packet decreases. Those results are totally different from the case of a highly coherent pulse train, such as a stable dissipative soliton laser, where the different frequencies contribute equally to the output pulses, and a filtering of output merely leads to broader pulse durations.

The loss of coherence is also reflected in the SOP of the filtered PML output. Figure 8 depicts the polarization states for different values of the wavelength and the pump power. Primarily, we observed that the SOP of the center wavelength remains as fixed point on the Poincaré sphere. On the other hand, Fig. 8 shows that wavelengths far away from the center mode bifurcate into a cross-like configuration of output SOPs. The two perpendicular lines in each cross are aligned with either a meridian or a parallel curve on the Poincaré sphere, respectively. This indicates that two separate processes are present that lead the azimuth and ellipticity angles of the output SOPs to evolve separately. The four directions of SOP evolution originate from vector FWM processes. Uneven fluctuations of the PI sidebands are responsible for stretching the corresponding SOP inside and outside of the cross. Besides, the two SOP curves along the parallel of the Poincaré sphere tend to bifurcate when a red-shifted wavelength component appears, such as in wavelength regions 5 & 6 for the pump power of 250 mW. This bifurcation of polarization may be determined by the coexistence of PMI-like process and XPMI-like process.

Furthermore, whenever the pump power reaches 250 mW, irregular polarization states located outside of the main polarization directions emerge for red-shifted sidebands. This SOP scattering aggravates over the whole wavelength span for a pump power of 450 mW. We attribute the polarization deviation from the cross shape to the chaotic competition of cascaded FWM processes, including both scalar and vector FWMs, through which the energy of longitudinal modes with a SOP along the cross is transferred into newly-generated SOPs. When the pump power is larger than 600 mW, additional longitudinal modes are populated, and the SOPs of filtered wavelengths appear as totally random, owing to enhanced spectral energy transfer processes. This randomization of the output SOP is associated with the loss of temporal coherence.

When considering the complex SOP evolutions in a PML, we envisage that such a light source can be utilized for highly coherent quantum entanglement applications. The all-fibre configuration makes such a source compatible with low loss optical networks. Moreover, frequencies with extreme intensities can be found at unpredictable positions on the broad spectrum. In analogy with incoherent spectral optical solitons^32^, our experiments indicate that that the loss of coherence in a PML could lead to the generation of incoherent spectral optical rogue waves. Such analysis will be the subject of subsequent detailed investigations. Given the analogies between the dynamics of PML and Kerr-combs, the analysis of spectra based on the DFT in our experiment could be helpful in the understanding the dissipative temporal and spectral pattern formation in microresonators, where is extremely hard to perform DFT detection due to the large bandwidth of the spectral output. Vector multi-wave-mixing processes could also be responsible for the formation of low coherence Kerr-combs in coherently pumped passive fiber cavities and microresonators. In these systems, vector modulation instabilities and parametric frequency conversion have been theoretically described^33^ and recently exploited for cross-polarized photon pair generation in a CMOS-compatible high refractive index glass integrated microresonator^34^. Thus we anticipate that our results could be beneficial for the understanding of the dynamics of coherence loss in Kerr combs and microcavity-based fiber laser systems^22^.

In summary, we experimentally investigated the dynamics of PML formation based on parametric instability followed by cascade FWM, controlled by polarization detuning. The PML first scatters its energy from a center wavelength mode into distant cavity modes via highly coherent parametric instability. Subsequently, all longitudinal modes are populated via cascaded FWM among the different orders of the parametric gain lobes. We found that mode interactions seeded by the highly coherent PI can either be competitive or cooperative, until eventually the laser system fully looses its coherence. In the incoherent stage, energy coupling among the various modes occurs when adjusting the intracavity SOP, and when increasing the external pumping strength, until a bifurcation into random SOPs is identified. The observed dynamics of spatio-temporal evolutions in a PML, leading to the loss of coherence, could find applications to supercontinuum and Kerr comb sources. These results may also provide a new perspective for the understanding of the loss of coherence in different nonlinear wave processes, even beyond the domain of nonlinear optics.

## Methods

### Measurement methods

The laser output is monitored by two kinds of detectors (PD1, 350 MHz; PD2, 50 GHz). The bandwidth of the oscilloscope is 1 GHz. As the rise time of PD1 is much larger than the duration of PML, the recorded pulse amplitude is proportional to the pulse energy. An autocorrelator with a delay resolution of 6 fs and an optical spectrum analyzer with a resolution of 0.02 nm are utilized. The SOP is measured by a high speed polarization measurement system. The real time oscilloscope in the DFT has a bandwidth of 20 GHz, and the signals are stretched by dispersive fibre with the dispersion of 300 ps^2^, and subsequently fed to a 50 GHz PD2 for frequency-to-time transformation. To ensure the detector is not saturated, a variable optical attenuator is inserted between the detector and the laser output.

### Numerical simulation

**Figure 1:**
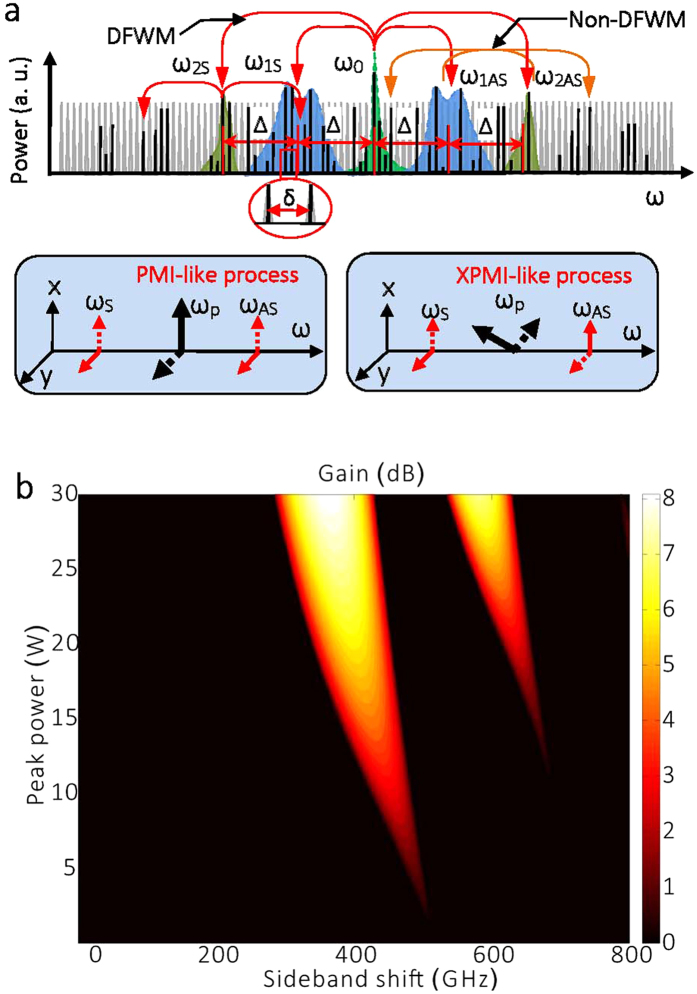
PML dynamics based on stochastic parametric conversion. (**a**) Primary gain sidebands, ω_1S_ and ω_1AS_, are generated from parametric instability: high-order gain lobes are activated with the optimization of the polarization state. Δ would be preserved in all sidebands due to energy conservation. Cascaded FWM populates longitudinal modes between the PI gain lobes. The insets are phase-matching processes of two different vector MI processes. The solid and dotted lines represent two orthogonal polarization components, respectively. In the PMI process, the polarizations of two sidebands are orthogonal to that of the pump, and are parallel to each other. In XPMI, sidebands are polarized at 45° from the pump laser polarization; (**b**) Numerically computed PI gain sidebands in the laser cavity with periodic dispersion and loss. The calculated sideband shift for the peak power of 5.15 W is about 490 GHz, which is close to the experimental value of 438 GHz. The discrepancy is mainly induced by the estimate error of the average power of pulses.

The parametric gain of Fig. 1(b) is computed from the Floquet method applied to the nonlinear Schrödinger equation (NLSE) with varying dispersion and nonlinear coefficients^35^. The evolution of the optical field *ψ* in fibre laser can be described by the NLSE that includes Kerr nonlinearity *γ* and second-order dispersion *β*_*2*_


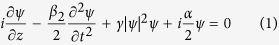


The influence of linear losses is included through the coefficient *α* (negative and positive values leading to distributed amplification and loss, respectively). In Eq. (1) we did not include higher-order dispersion or Raman scattering. These effects do not have a noticeable influence on the MI spectral dynamics in the fibre laser. With the change of variables *U* = *ψ* exp(*α*z/2), one obtains from (1)


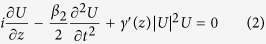


where *γ*′ = *γ* exp(−*α*z). In the simulations, we consider a Kerr nonlinearity of *γ* = 2 W^−1^km^−1^, a spatial period of the dispersion variation equal to the cavity length (35 m), comprising 19.5 m span of DCF (with dispersion *D* = −38 ps/nm/km and loss of 0.5 dB/km), 14.5 m span of SMF (with dispersion *D* = 18 ps/nm/km and loss of 0.2 dB/km), and 1 m EDF (with dispersion *D* = 15.7 ps/nm/km and a gain that compensates cavity loss exactly). Before the EDF, we inserted a 1dB of lumped loss to take into account out-coupling loss and connector losses. The pulse duration of PC state 1 is about 0.04 ns. According to the relative pulse energy value detected by low speed PD1, the average power of the pulses in the cavity is about 1.2 mW. Thus, the peak power is about 5.15 W. We may write the perturbed CW solution of the NLSE (2) as


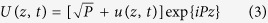


where we suppose *|u|*^*2*^<<*P*, so that one obtains the linearized equation for *u*(*Z, T*)


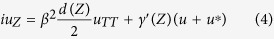


where we define *β*^*2*^ = *L*_*nl*_*/L*_*d*_, (the nonlinear length *L*_*nl*_ = *1/*(*γP*), the dispersion length *L*_*d*_ = *t*_*0*_^*2*^*/|β*_*2ave*_*|,* and the reference time unit *t*_*0*_ = *1 ps*). Therefore, *Z* = *z/L*_*nl*_, and *T* = *t/t*_*0*_. By writing the solution of (4) as the sum of Stokes and anti-Stokes sidebands, *u*(*Z,T*) = *a*(*Z*) exp*{i*Ω*T*)+b^*^(*Z*) exp{−*i*Ω*T*), we obtain two coupled linear ordinary differential equations (ODEs) with periodic coefficients for *a*(*Z*) and *b*(*Z*)


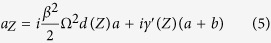



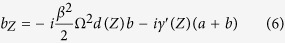


Equations (5) and (6) are equivalent to a single, second-order ODE, and a linear stability analysis can be rigorously carried out numerically by the Floquet theory, which is analogous to Bloch wave theory in solid state physics. By defining the solution vector of (5) and (6) as ***s*** = (*a*, *b*), and choosing the two fundamental initial conditions ***s***_*1*_(*Z* = *0*) = (*1, 0*) and ***s***_*2*_(*Z* = *0*) = (*0*, *1*), we obtains the principal solution matrix S = [**s**_1_(*L*′)^t^, **s**_2_(*L*′)^t^] (where *t* denotes the vector or matrix transpose) from the solutions of (5) and (6) at *Z* = *L*′ (where *L*′ = *L*/*L*_*nl*_). According to Floquet’s theorem, the eigenvalues of S, or Floquet’s multipliers λ = exp (η_*F*_ + *i*σ), such that |λ|>1 yields the linear instability of the CW with respect to the growth of sidebands with frequency detuning Ω. Since the scattering matrix after an integer number n of periods is simply S^n^, the Floquet’s multipliers to the MI gain G after one period of the dispersion and power oscillation in the laser cavity can be related by G = 2η_F_/*L*′.

## Additional Information

**How to cite this article**: Gao, L. *et al.* Coherence loss of partially mode-locked fibre laser. *Sci. Rep.* **6**, 24995; doi: 10.1038/srep24995 (2016).

## Supplementary Material

Supplementary Information

## Figures and Tables

**Figure 2:**
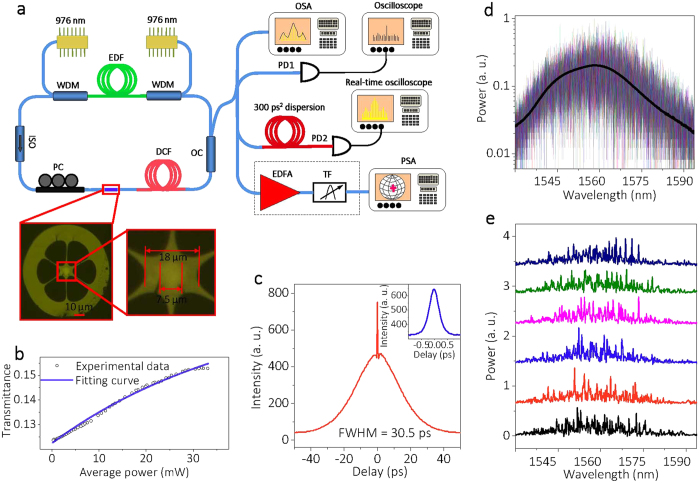
Laser setup and typical outputs for pump power at 800 mW. (**a**) EDF, erbium-doped fibre (Liekki ER 80-8/125); WDM, wavelength division multiplexer; ISO, polarization independent optical isolator; PC, polarization controller; DCF, dispersion compensation fibre; OC, optical coupler; OSA, optical spectrum analyzer; PD, photo-detector; EDFA, erbium-doped fibre amplifier; TF, tunable filter; PSA, polarization state analyzer. The inset is the cross section of the PCF; more details are given in the Supplementary. (**b**) Nonlinear transmission of the SA as a function of average power. (**c**) Autocorrelation trace: the inset is the coherent peak in a larger scale. (**d**) 100 single-shot spectra measured by DFT (colored lines), and the black line is the averaged spectrum measured with an OSA. (**e**) 6 consecutive single-shot spectra showing enhanced spectral fluctuations in PML.

**Figure 3:**
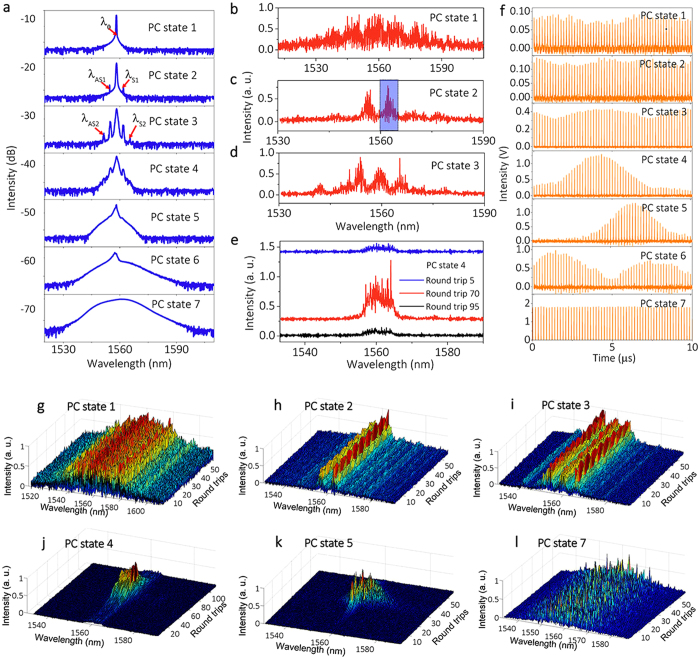
Evolution of PML with polarization detuning. (**a**) Averaged optical spectra for different PC states. The center wavelength λ_0_ is 1558.43 nm, and the four new wavelengths are λ_2AS_ = 1551.4 nm, λ_1AS_ = 1554.8 nm, λ_1S_ = 1561.6 nm, λ_2S_ = 1565.1 nm. (**b**–**e**) Corresponding single-shot spectra for different PC states in (**a**). (**f**) Corresponding temporal pulse train detected by PD1. (**g**–**l**) Consecutive single-shot spectra under PC states 1 to 5 and 7.

**Figure 4:**
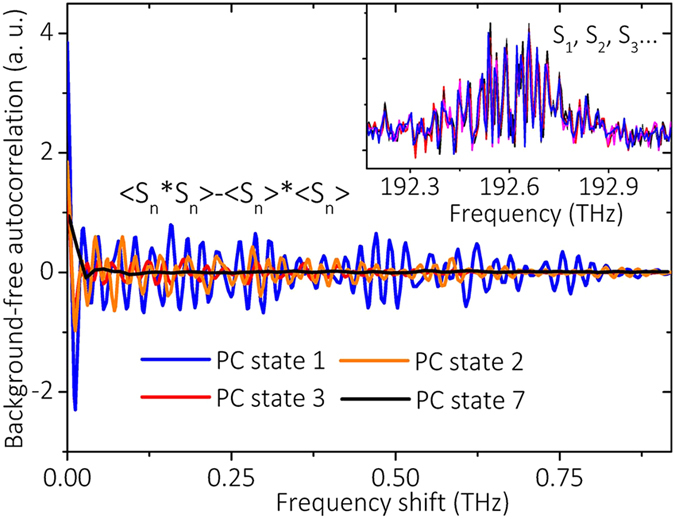
Autocorrelation analysis of the first Stokes region in single-shot spectra for different PC states. The inset contains 5 consecutive single-shot spectra within the first Stokes region (shaded region in Fig. 3(c)) under PC state 2, where only small fluctuations are shown.

**Figure 5:**
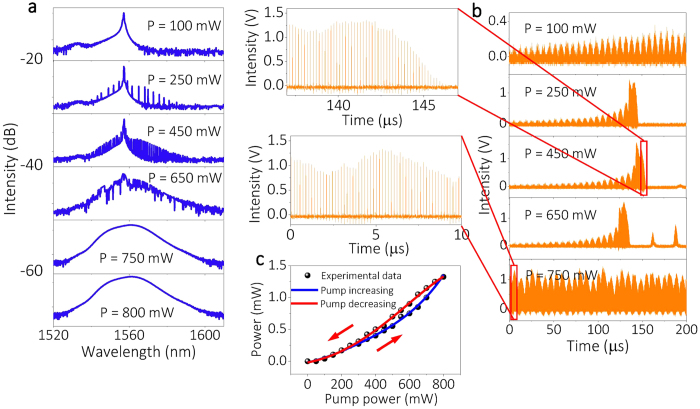
Evolution of PML as the pump strength is varied. (**a**) Averaged optical spectra for different pump power levels. (**b**) Corresponding temporal traces detected by PD1. The insets illustrate the cavity output over a smaller time range. (**c**) Hysteresis effect of average output power under different pumping regimes (increasing vs. decreasing pump power).

**Figure 6:**
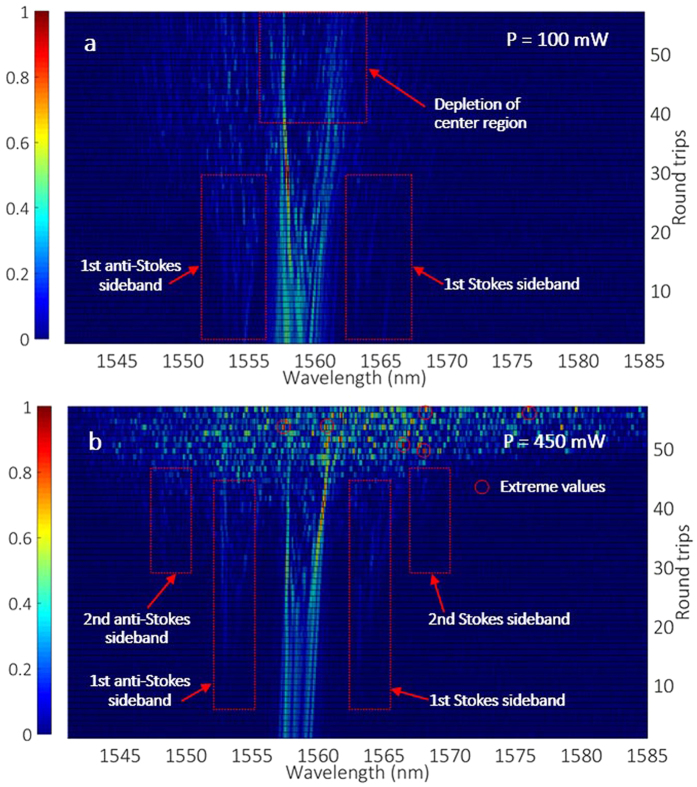
Single-shot spectra for pump powers of (**a**) 100 mW or (**b**) 450 mW, respectively. Regions dotted by red circles indicate frequencies with extreme intensities, which could be associated with the presence of spectral optical rogue waves.

**Figure 7:**
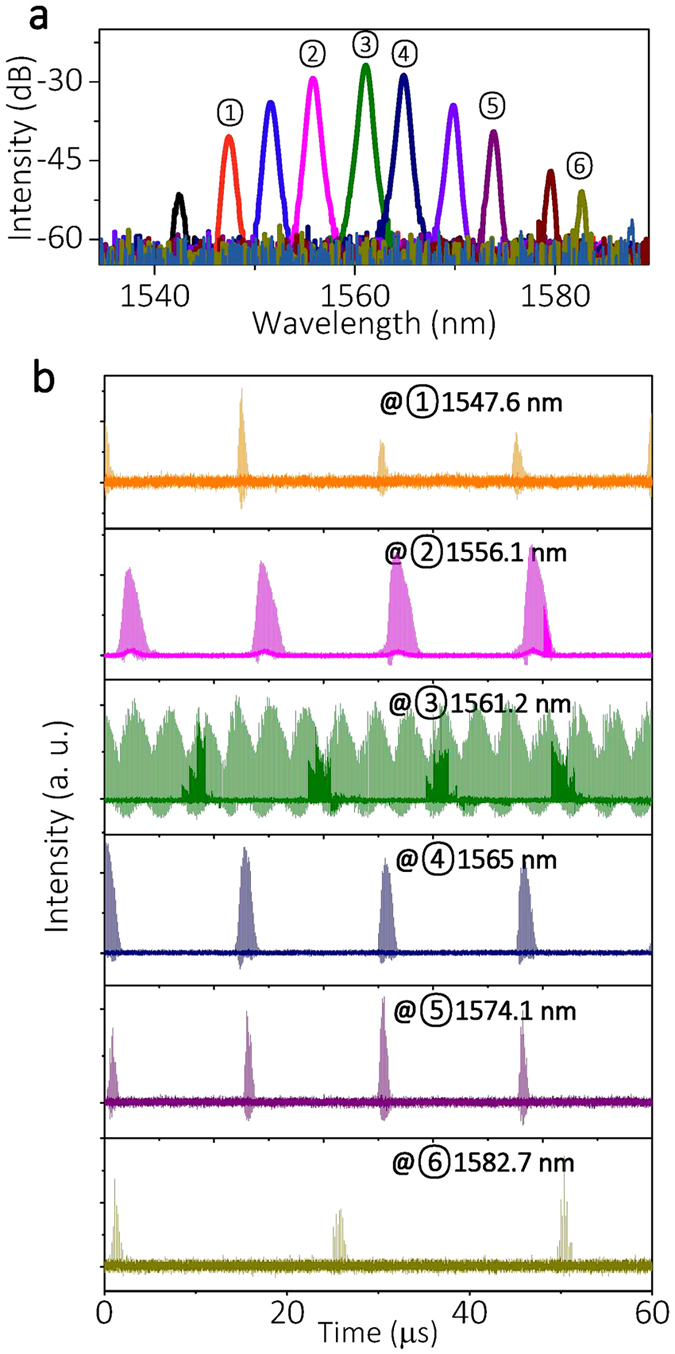
(**a**) Filter spectra with different center wavelengths, and (**b**) corresponding temporal traces. Index (3) indicates the central spectral region, while (2) and (4) indicate the primary Stokes and anti-Stokes gain lobes.

**Figure 8:**
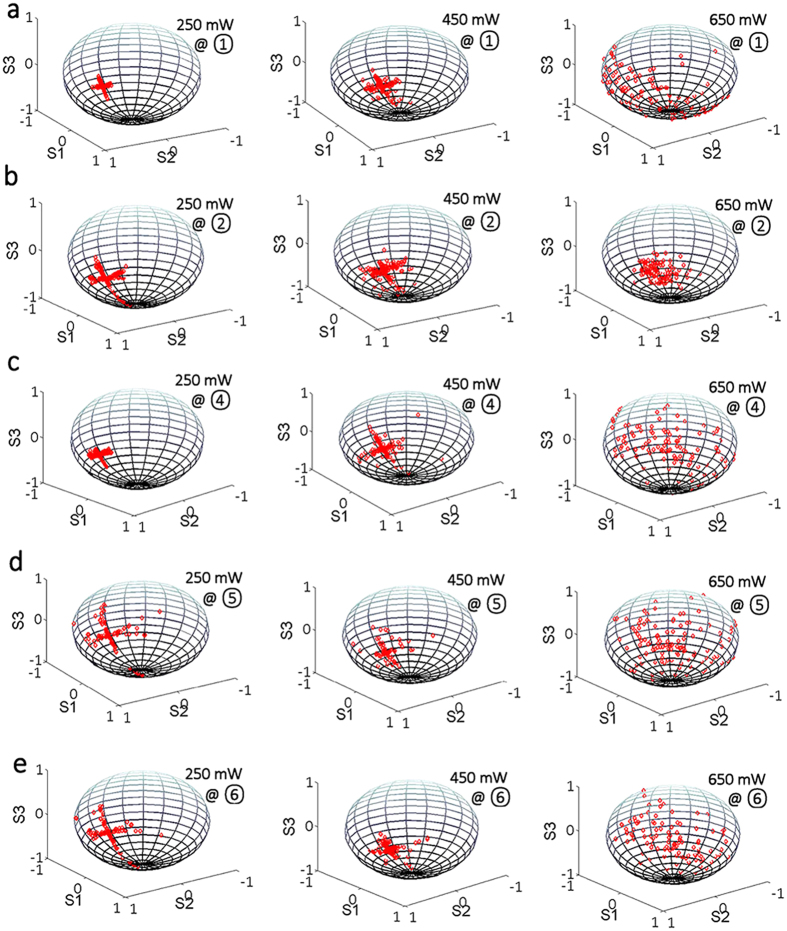
Experimentally measured polarization states for filtered wavelengths under various pump powers. The polarization states of center wavelength at (3) are always as a fixed point in the Poincaré sphere for different pump powers (not shown).
