# Supplementary material for: Coherence loss of partially mode-locked fibre laser
Source: Sci Rep. 2016 Apr 29;6:24995. doi: 10.1038/srep24995 (PMC4850403; doi:10.1038/srep24995)
Supplement: Supplementary Information [file srep24995-s1.doc]

Supplementary Information

Coherence loss of partially mode-locked fibre laser

Lei Gao^1^, Tao Zhu^1*^, Stefan Wabnitz^2^, Min Liu^1^, and Wei Huang^1^

^1^Key Laboratory of Optoelectronic Technology & Systems (Ministry of Education), Chongqing University, Chongqing 400044, China.

^2^Dipartimento di Ingegneria dell’Informazione, Università degli Studi di Brescia and INO-CNR, via Branze 38, 25123 Brescia, Italy.

Corresponding email: zhutao@cqu.edu.cn

1 Fabrication of saturable absorber

The reduced graphene oxide (rGO) used in our experiment is prepared by reducing chemical oxidation of graphite. Similar to graphene, rGO has also been intensively investigated for mode-locked laser applications, due to the Pauli blocking when it is excited by strong light with single-photon energy larger than the energy gap [1, 2]. Moreover, rGO disperses well in water for functional groups such as hydroxyl and epoxide, on its sheet edges, and the size of the rGO particles can be manipulated. The thickness of the rGO here is about 0.55–3.74 nm, corresponding to 2-10 number of layers. We depict the Raman spectrum of the rGO sample in Fig. S1 (a), where two major peaks of D band and G band are located at∼1320 cm^−1^ and ∼1610 cm^−1^, respectively. Although this rGO has a large defect density, the defect contribution to the mode-locking process can be neglected.

The saturable absorber (SA) is produced by filling rGO dissolved in N,N-dimethylformamide into the holes of the PCF. Primarily, we produce the transparent rGO solution through centrifuging the ultrasound-treated rGO-N,N-dimethylformamide solution. Then, we fill the cladding holes of the photonic crystal fibre (PCF) with the rGO solution based on Siphon Effect, and the filled PCF is dried in a vacuum chamber at 38° for 24 hours to avoid the rGO particles movement in solution. Finally, 2 cm PCF is spliced between single mode fibres (SMF, Corning SMF-28) with a splice loss of 3 dB, which is mainly induced by mode field mismatch.

The PCF contains an inner core and inner cladding with diameters of 7.5 μm and 18 μm, respectively. Figure S1 (b) depicts the intensity distribution of the fundamental mode, where light intensity vs fibre radius reveals that only 1/10^7^ of light passing through the PCF will interact with rGO. This ultraweak interaction intensity enhances the thermal damage threshold and correspondingly decreases the nonlinearity.

The transmission of the SA is detected by an optical spectrum analyzer (Si720, Micro Optics) with an inner tunable laser source with a power of 1 mW, and the experimental result is shown in Fig. S 1 (c). We find that the absorption induced by rGO flakes of the 2 cm PCF is about 5.8 dB, which is consistent with the nonlinear transmission in Fig. 2 (b) in the main manuscript. The polarization-dependent loss (PDL) of the pure PCF spliced with SMF is about 0.2 dB, while it increased to ~3 dB when the PCF is filled with polarization-dependent rGO flakes, which are shown in Fig. S1 (d).


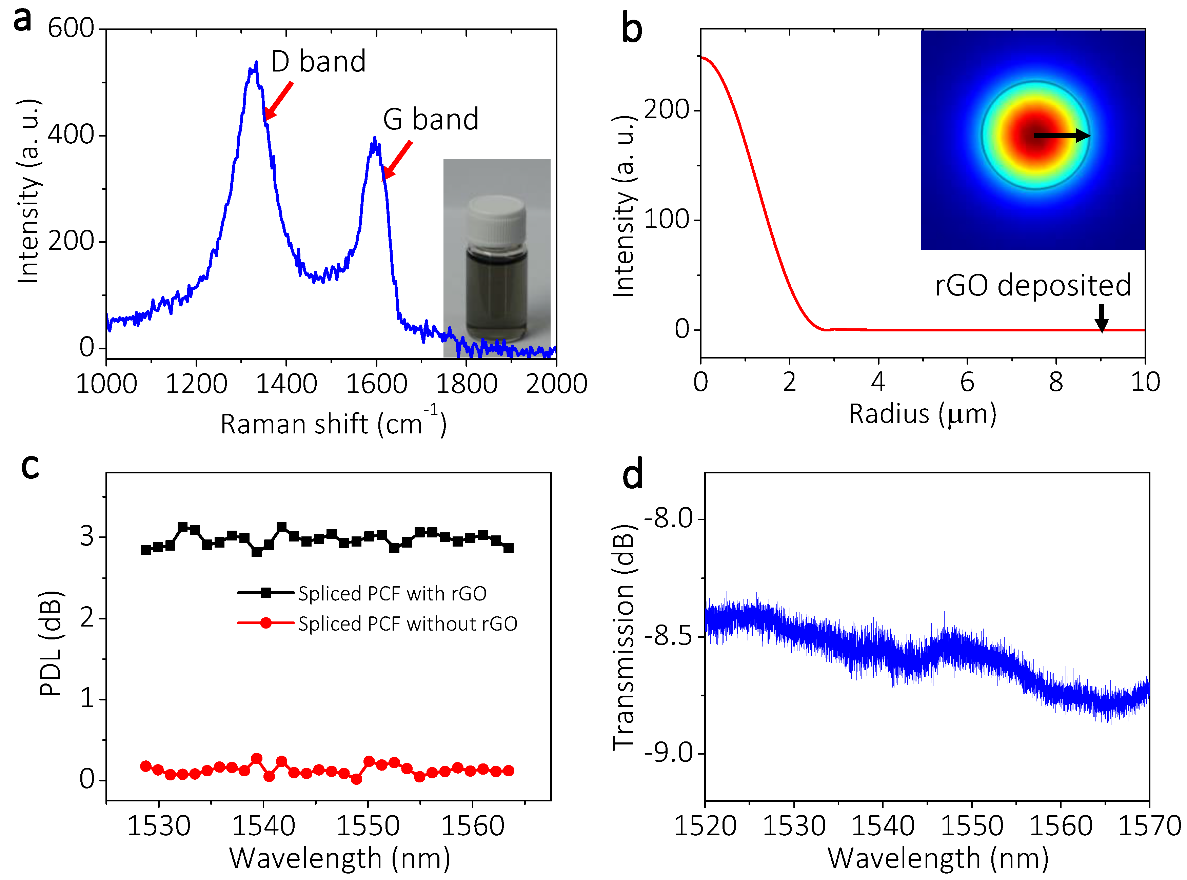


**Figure S1. Characteristics of the SA.** (a) Raman spectrum of the rGO sample; the inset shows the transparent solution of rGO and N,N-dimethylformamide. (b) Light intensity vs fibre radius, and the intensity distribution of LP_01_ mode are represented in the inset. (c) Transmission spectrum of the fabricated SA. (d), PDLs of spliced PCF with/without rGO flakes.

The nonlinear optical response of the fabricated SA is detected by means of a balanced two-detector method as in [3]. The 10% port of the coupler is utilized as the reference arm, while the other arm is fused with the SA for testing. A variable optical attenuator is used to change the average power, and two identical power meters are utilized. The pulse source is a home made ultrafast fibre laser centered at 1563 nm, with pulse duration and repetition rate of about 285 fs and 7.4 MHz, respectively. As the rGO flakes are only partially interacting with the PCF, the effective interacting area is hard to estimate. Here, we use the pulse power rather than the pulse intensity to present the nonlinear response. The nonlinear transmission of Fig. 2 (b) is fitted by a typical nonlinear transmission formula [3]. The calculated values for saturable power, transmittance difference, and the nonsaturable absorption are 15 mW, 3% and 0.66 mW, respectively. As the transmittance loss is about 8.8 dB, the modulation depth is about 24%.

2 Effects of SA and dispersion compensation fibre in forming partially mode-locked fibre laser

We checked the effect of rGO in the formation of a partially mode-locked fibre laser (PML). When the rGO is replaced by carbon nanotubes or topological insulator Bi_2_Se_3_ nanosheets, similar results are obtained. These results indicate that the nonlinear phase of a SA is crucial for generating the PML, irrespective of which kind of nonlinear material is used.

We experimentally studied the effects of SA and DCF in forming PML. Once the SA is removed, neither PML nor conventional soliton could be found. When the DCF is removed from the cavity, no PML but only conventional soliton can be found. When the normal dispersion is decreased to 0.1 ps^2^, only dissipative soliton arises. When 500 m SMF is added to the original cavity, only PML could be found.

3 Autocorrelation analysis of single-shot spectra


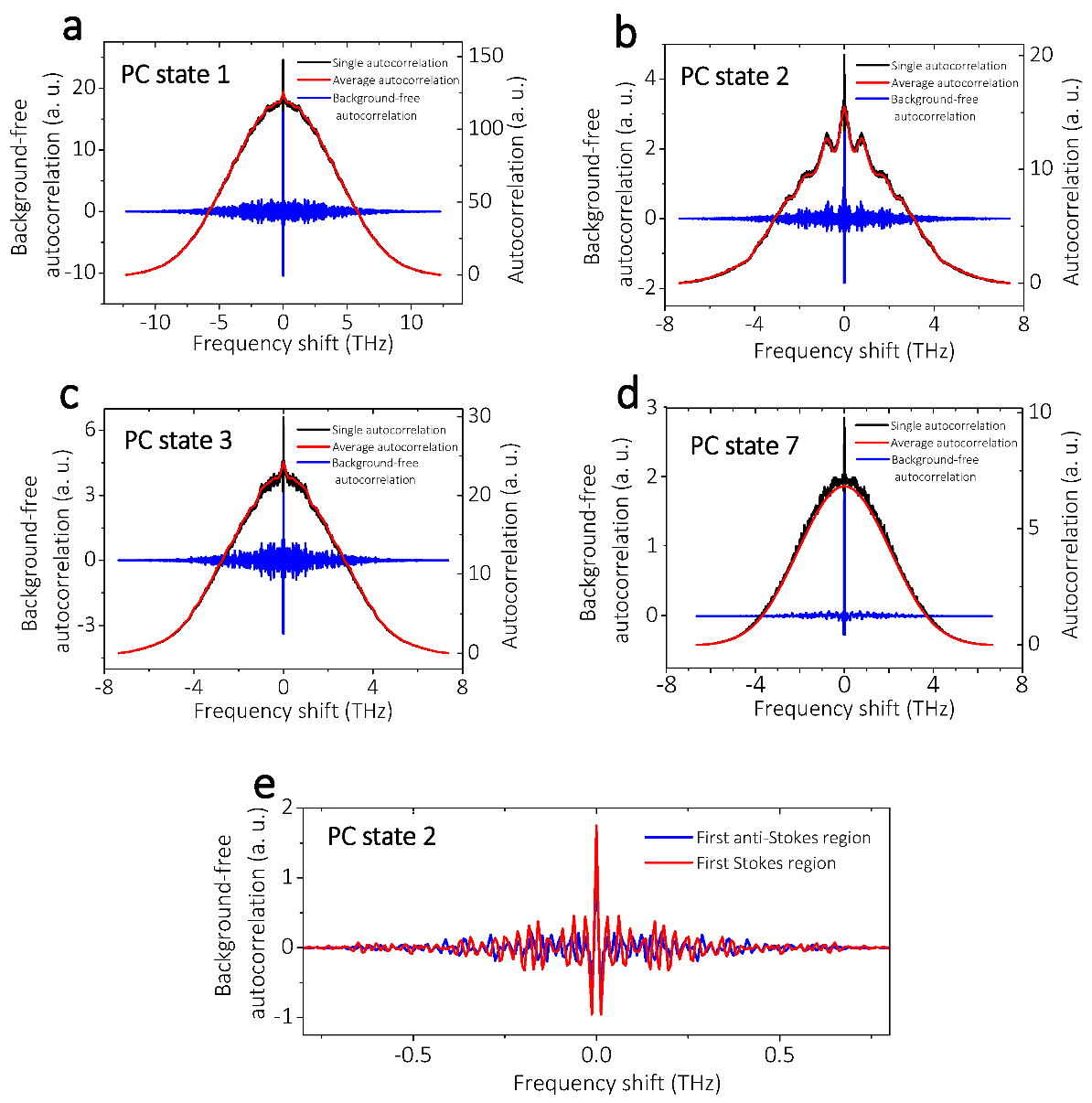


**Figure S2. Autocorrelation analysis of single-shot spectra for different PC states.** (a)-(d) Background-free autocorrelations, single autocorrelation and average autocorrelation of the whole single-shot spectra for PC states 1, 2, 3, and 7. (e) Background-free autocorrelations of the first anti-Stokes and Stokes regions of PC state 2.

We plot the background-free autocorrelations, together with a single autocorrelation and an average autocorrelation within the whole single-shot spectra for different PC states in Fig. S2 (the calculation method is described in the main text). It is clear that the quasi-periodic cooperative and competitive mode interactions of the primary Stokes gain bands shown in the main text are absent when taking the autocorrelation over the whole spectrum. However, we can still find that finer structures are present in the initial PC states, especially for PC state 2, and this autocorrelation diminishes to a small value when PML is formed. This loss of correlation is in accordance to the conclusion in the main text that the formation of PML is accompanied with a loss of coherence.

We compare the coherence of the anti-Stokes and the corresponding Stokes region based on background-free autocorrelations. As shown in Fig. S2 (e), both the difference of intensity and quasi-period of the correlation values are depicted. The intensity difference is mainly induced by the symmetry-breaking of the MI spectrum [4], due to the third-order dispersion. The slight difference of the quasi-periods of the MI bands in the anti-Stokes and Stokes regions may be attributed to the different dispersions of the dispersion fibre in the disperse Fourier transformation detection at different wavelengths.

1. Sobon, G. *et al*. Graphene oxide vs. reduced graphene oxide as saturable absorbers for Er-doped passively mode-locked fiber laser. *Opt. Express* **20**, 19463-19473 (2012).
2. Lin, Y. H. *et al*. Using graphene nano-particle embedded in photonic crystal fiber for evanescent wave mode-locking of fiber laser. *Opt. Express* **21**, 16763-16776 (2013).
3. Gao, L. *et al*. Stable, ultrafast pulse mode-locked by topological insulator Bi2Se3 nanosheets interacting with photonic crystal fiber: from anomalous dispersion to normal dispersion. *IEEE Photon. J.* **7**, 3300108 (2015).
4. Droques, M. *et al*. Symmetry-breaking dynamics of the modulational instability spectrum. *Opt. Lett*. **36**, 1359-1361 (2011).
